# SerpinB3 and Yap Interplay Increases Myc Oncogenic Activity

**DOI:** 10.1038/srep17701

**Published:** 2015-12-04

**Authors:** Cristian Turato, Stefania Cannito, Davide Simonato, Gianmarco Villano, Elisabetta Morello, Liliana Terrin, Santina Quarta, Alessandra Biasiolo, Mariagrazia Ruvoletto, Andrea Martini, Silvano Fasolato, Giacomo Zanus, Umberto Cillo, Angelo Gatta, Maurizio Parola, Patrizia Pontisso

**Affiliations:** 1 Dept. of Medicine, University of Padova, Italy; 2 Dept. of Clinical and Biological Sciences, Unit of Experimental Medicine and Interuniversity Center for Liver Pathophysiology, University of Torino, Italy; 3 Unit of Hepatobiliary Surgery and Liver Transplantation, University of Padova, Italy

## Abstract

SerpinB3 has been recently described as an early marker of liver carcinogenesis, but the potential mechanistic role of this serpin in tumor development is still poorly understood. Overexpression of Myc often correlates with more aggressive tumour forms, supporting its involvement in carcinogenesis. Yes-associated protein (Yap), the main effector of the Hippo pathway, is a central regulator of proliferation and it has been found up-regulated in hepatocellular carcinomas. The study has been designed to investigate and characterize the interplay and functional modulation of Myc by SerpinB3 in liver cancer. Results from this study indicate that Myc was up-regulated by SerpinB3 through calpain and Hippo-dependent molecular mechanisms in transgenic mice and hepatoma cells overexpressing human SerpinB3, and also in human hepatocellular carcinomas. Human recombinant SerpinB3 was capable to inhibit the activity of Calpain *in vitro*, likely reducing its ability to cleave Myc in its non oncogenic Myc-nick cytoplasmic form. SerpinB3 indirectly increased the transcription of Myc through the induction of Yap pathway. These findings provide for the first time evidence that SerpinB3 can improve the production of Myc through direct and indirect mechanisms that include the inhibition of generation of its cytoplasmic form and the activation of Yap pathway.

Primary liver cancer, mainly hepatocellular carcinoma (HCC), is one of the leading causes of cancer-related death worldwide^1^ and generally has a poor prognosis since it is often diagnosed at an advanced stage when treatment is not effective. Great efforts have been made to decipher the molecular mechanisms of HCC and gene expression profiling has been used to identify subgroups of patients according to etiological factors, early pre-neoplastic lesions, stage of the disease, rate of recurrence and survival^2^,^3^,^4^,^5^. Genetic analysis has revealed that Myc oncogene over-expression is present in up to 70% of viral and alcohol-related HCCs. Myc is a potent activator of tumorigenesis and its deregulation has been shown to contribute to a variety of cancers^6^,^7^,^8^. Increased Myc levels often correlate with the more advanced and aggressive tumour forms, providing a characteristic signature of cancer progenitor cells and suggesting that its overexpression plays some part in Myc-driven pathogenesis and tumor biology^9^,^10^.

The Myc oncoprotein is a pleiotropic transcription factor belonging to the basic helix-loop-helix-leucine zipper family with a critical role in engaging and coordinating expression of the genes necessary for efficient and ordered proliferation of somatic cells. Myc expression is highly regulated being tightly controlled by a number of mechanisms involving many transcriptional regulatory motifs^11^,^12^. According to its well established predominant role Myc regulates up to 15% of human genes and is involved in cell growth, proliferation, metabolism, differentiation, and apoptosis^6^.

According to data emerging from genome-wide gene expression profiling^13^ Myc has been recently suggested to be at the centre of human liver tumor malignant conversion.

As expected, given their broad role as transcriptional regulator, Myc family proteins are predominantly localized within nuclear compartment during proliferation, although emerging data indicate that Myc biology may be even more complex. Accordingly, a recent finding suggests that the cysteine protease Calpain can convert Myc into Myc-nick, a calpain cleavage product which lacks the nuclear localization signal and DNA-binding domain, regulating alpha-tubulin acetylation and promoting cell differentiation^14^.

Along these lines, the Yes-associated protein (Yap) is a critical regulator of proliferation, liver size and self-renewal of normal and cancer stem cells^15^. This oncogene is the main effector of the Hippo pathway, and it is up-regulated in almost 62% of HCC tissues^16^. Yap protein is usually detected at low level in differentiated hepatocytes, with the signal being diffuse throughout the cell^17^, but in poorly differentiated cancer cells high levels of Yap can be detected within the nuclear compartment. Pertinent to this study, recent data suggest that Yap can exert part of its oncogenic activities in liver cancer cells also through the modulation of Myc^18^.

SerpinB3, a member of the ovalbumin-serine proteases inhibitor family (ov-serpins)^19^, has been detected in several malignancies of epithelial origin, including HCC^20^,^21^,^22^,^23^. In the liver, SerpinB3 is almost undetectable in normal hepatocytes but it is progressively up-regulated in liver cirrhosis, dysplastic nodules and hepatocellular carcinoma, suggesting that this serpin may be involved in the early events of hepatocarcinogenesis^24^,^25^.

This serpin determines apoptosis resistance^26^,^27^ and induces cell proliferation and deregulation of adhesion processes, leading to epithelial-to-mesenchymal transition (EMT) and increased invasiveness^28^. Moreover, transgenic mice over-expressing SerpinB3 showed higher liver regenerative capacity compared to wild-type mice, suggesting a role of this protein in promoting cell growth^29^. Recent studies indicate that SerpinB3 is up-regulated by oncogenic Ras, promotes NF-kB-related inflammatory cytokine production^30^ and induces IL-6 autocrine signaling^31^, favouring tumour progression. These findings are in keeping with the reported high levels of SerpinB3 detected in the sub-class of HCCs with poor prognosis^32^.

A previous study from our group has shown that SerpinB3 is expressed in the majority of hepatoblastomas, where SerpinB3 levels were positively correlated with the extent of Myc expression and tumour diffusion^33^. Although SerpinB3 has been described as an early marker of liver carcinogenesis, the potential mechanistic role of SerpinB3 as a mediator of HCC development is still poorly understood. Since this serpin is emerging as a relevant hinge for the aggressive phenotype of primary liver cancers and Myc overexpression is associated with poor prognosis, the working hypothesis is that SerpinB3 might be involved in the deregulation of Myc oncogene and that its relationships with Myc expression may be critical for liver carcinogenesis.

## Results

### Expression and localization of Myc in relation to SerpinB3

**Figure 1:**
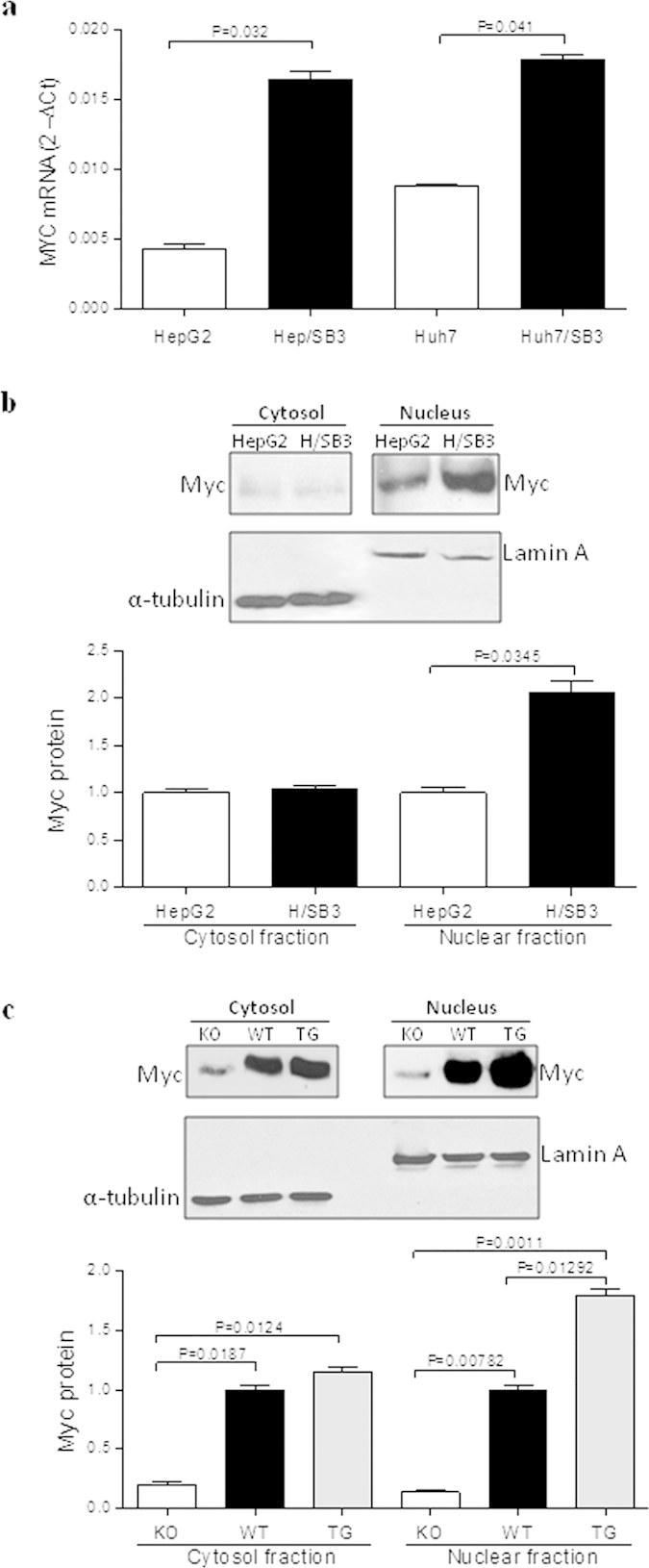
Expression, subcellular localization of Myc. (**a**) mRNA expression of Myc in HepG2 and Huh7 cells stably transfected with SerpinB3 (/SB3) or with the empty vector, as control (HepG2 or Huh7). Changes in mRNA gene expression were reported using the 2-ΔCT method. Values represent normalized mean expression ±  SEM of three independent experiments performed in triplicate. (**b**) Representative Myc western blot analysis and densitometric analysis results of three independent experiments performed on cytoplasmatic and nuclear extracts in control HepG2 cells and HepG2 cells transfected with SerpinB3 (H/SB3). Values represent mean normalized expression ± SEM. (**c**) Representative western blot and densitometric analysis results of three independent experiments performed in SerpinB3-knockout mice (KO), in wild type C57BL/6 mice (WT) and in mice transgenic for human SerpinB3 (TG). The cropped gels shown in this figure have been run under the same experimental conditions and the values represent mean normalized expression  ± SEM of three independent experiments.

**Figure 2:**
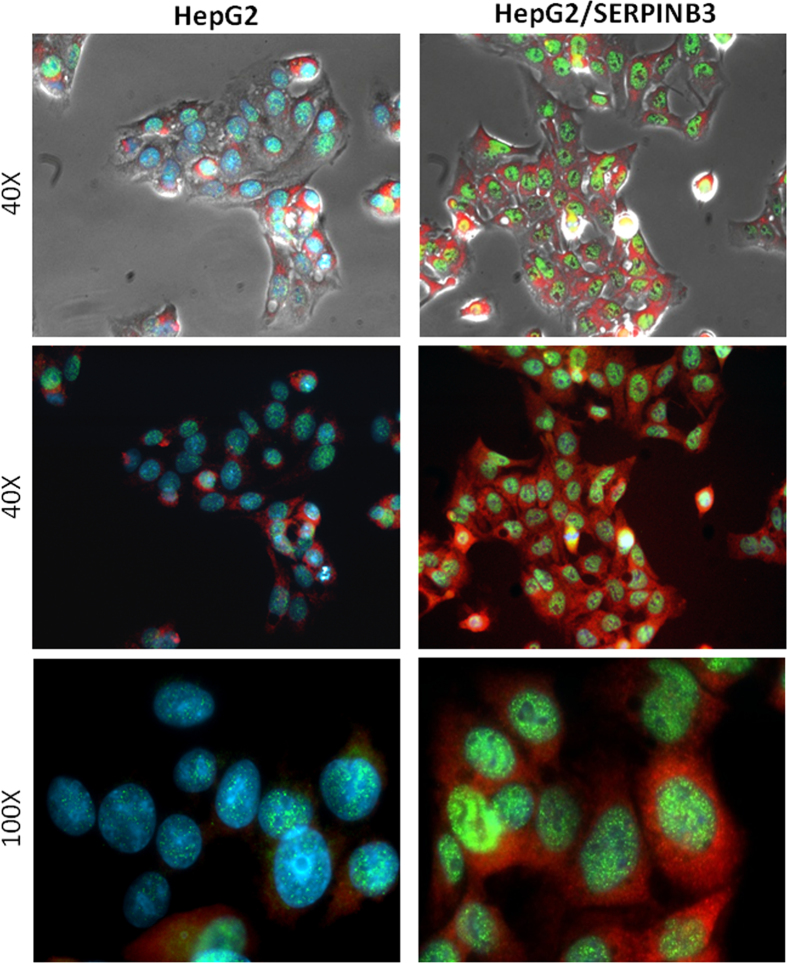
Immunoflorescence results. Merge of contrast phase (first row) and indirect immunofluorescence (second and third row) analysis for SerpinB3 (red, Cy3) and Myc (green, 488) performed in control HepG2 and in HepG2 cells overexpressing SerpinB3 (HepG2/SERPINB3). Nuclei are stained in blue (dapi). Original magnification are reported. Evaluation of immunoflorescence was assessed in three independent experiments examining at least ten random high-power fields.

In order to investigate whether SerpinB3 has a role in Myc regulation, the expression of Myc was explored in different models. In both HepG2 and Huh7 cells stably transfected to overexpress SerpinB3, Myc transcript levels were found significantly increased, as compared to parental cells (Fig. 1a). At protein level, subcellular analysis performed in transfected HepG2 cells versus parental cells revealed a pronounced nuclear increase, as documented by Western blot (Fig. 1b) and by immunofluorescence (Fig. 2) analysis, respectively.

**Figure 3:**
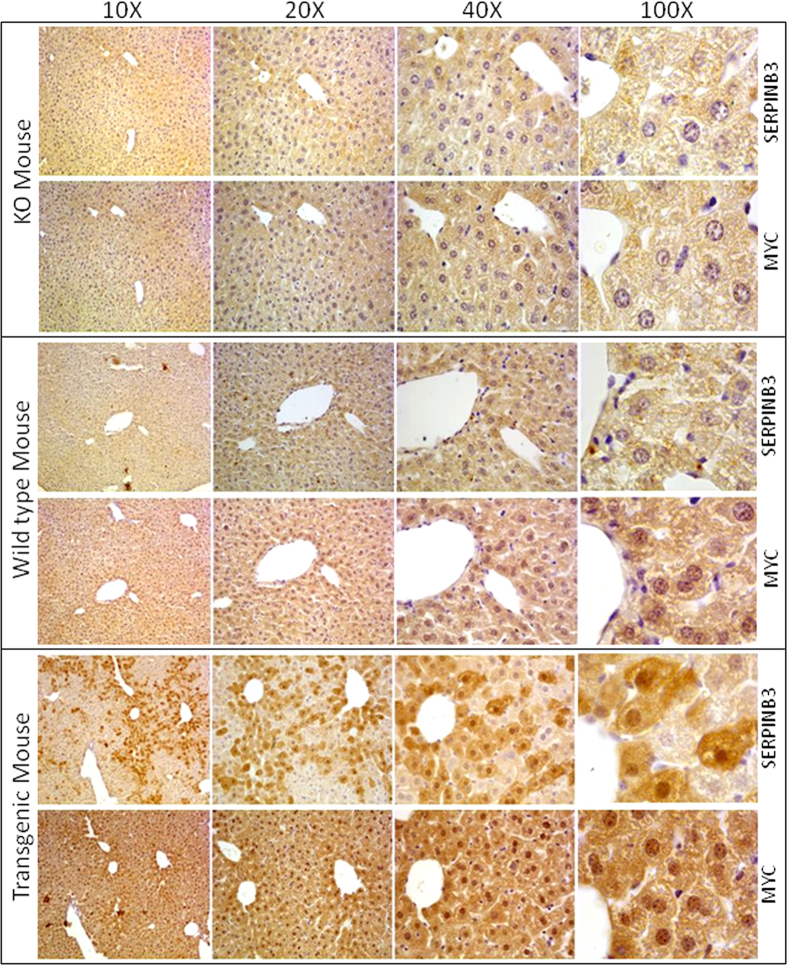
Immunohistochemistry in mice liver. Representative image of immunohistochemistry for SerpinB3 and Myc in serial liver sections of SerpinB3-knockout mice (KO), wild type mouse and SerpinB3 transgenic mice. Original magnifications are reported. Evaluation of immunostaining was assessed by two investigators examining at least ten random high-power fields.

Myc expression was then assessed in liver specimens obtained from C57BL6 transgenic mice overexpressing SerpinB3 and from Serpinb3a(−/−) mice. SerpinB3 transgenic mice showed a significant increase of nuclear Myc protein levels versus respective controls as evaluated in the representative Western blot analysis of Fig. 1c. Results obtained in the additional animals analyzed is shown in Supplemental Fig. 1. Immunohistochemistry confirmed the higher expression of nuclear Myc localization in SerpinB3 transgenic mice (Fig. 3). Conversely, in SerpinB3a(−/−) mice the levels of Myc where significantly lower than those of control littermates, both in the cytoplasm and in the nucleus (Figs 1c and 3), overall suggesting a predominant role of SerpinB3 in the regulation of Myc oncogene expression.

**Figure 4:**
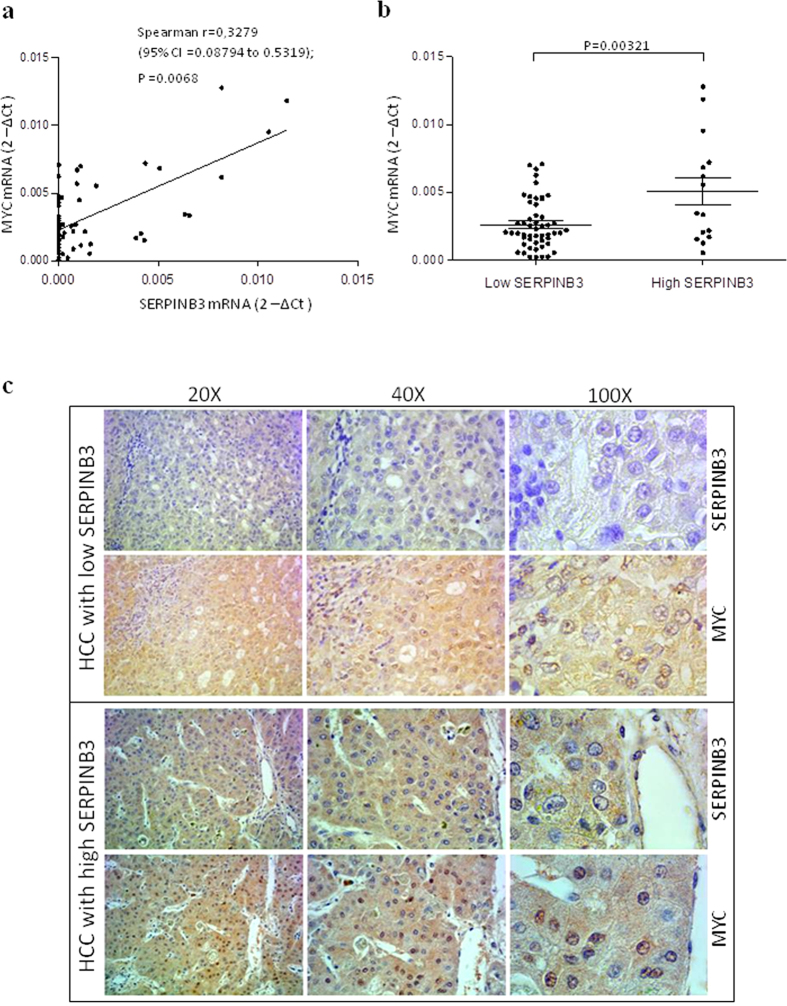
Myc and SerpinB3 in hepatocellular carcinoma. (**a**) Relationship between SerpinB3 and Myc mRNA in human HCCs. The axis represents the relative mRNA amount of the normalised genes, calculated by dividing the non-normalized values by the housekeeping genes and expressed as arbitrary units. The correlation analysis was performed with Spearman r test. (**b**) mRNA in human HCCs. Myc mRNA was analysed by qRT-PCR in relation to low expression (<median value; N = 52) or high expression (>median value; N = 15) of SerpinB3 mRNA. Central bars represent mean and external bars represent S.E.M. Statistical analysis was performed using Mann Whitney test. (**c**) Representative examples of immunohistochemistry for SerpinB3 and Myc counterstained with hematoxylin, in serial sections of a human HCC specimen with low expression of SerpinB3 and of a human HCC with high expression of SerpinB3. Original magnifications are reported.

These results were in full agreement with findings obtained in human HCC specimens from a cohort of well characterized patients. Myc mRNA levels were detectable in 64% of the cases and showed a positive correlation with SerpinB3 transcript levels (Fig. 4a). The highest levels of Myc transcription were found in the sub-class of patients with high (>median value) SerpinB3 expression (Fig. 4b). Moreover, immunohistochemical analysis in serial paraffin sections revealed a prevalent nuclear localization of Myc in HCC areas showing high positivity for SerpinB3, while prevalent cytoplasmic localization was observed in HCC areas showing no/low positivity for SerpinB3, as shown in Fig. 4c. Despite these findings, high Myc expression did not show significant clinical association with early tumor recurrence (p value = 0.3677), as previously described for SerpinB3 in the same cohort of patients^32^. In addition, by univariate and multivariate analysis, SerpinB3 and the severity of liver cirrhosis (identified with class B and C of CHILD-PUGH score^34^), but not Myc, were identified as independent variables associated to early HCC recurrence (Supplemental Table 1). These findings suggest a primary involvement of SerpinB3 in hepatocarcinogenesis, while the presence of Myc might also enhance tumor aggressiveness and invasiveness potential.

### SerpinB3 promotes mRNA expression of Myc target genes

**Figure 5:**
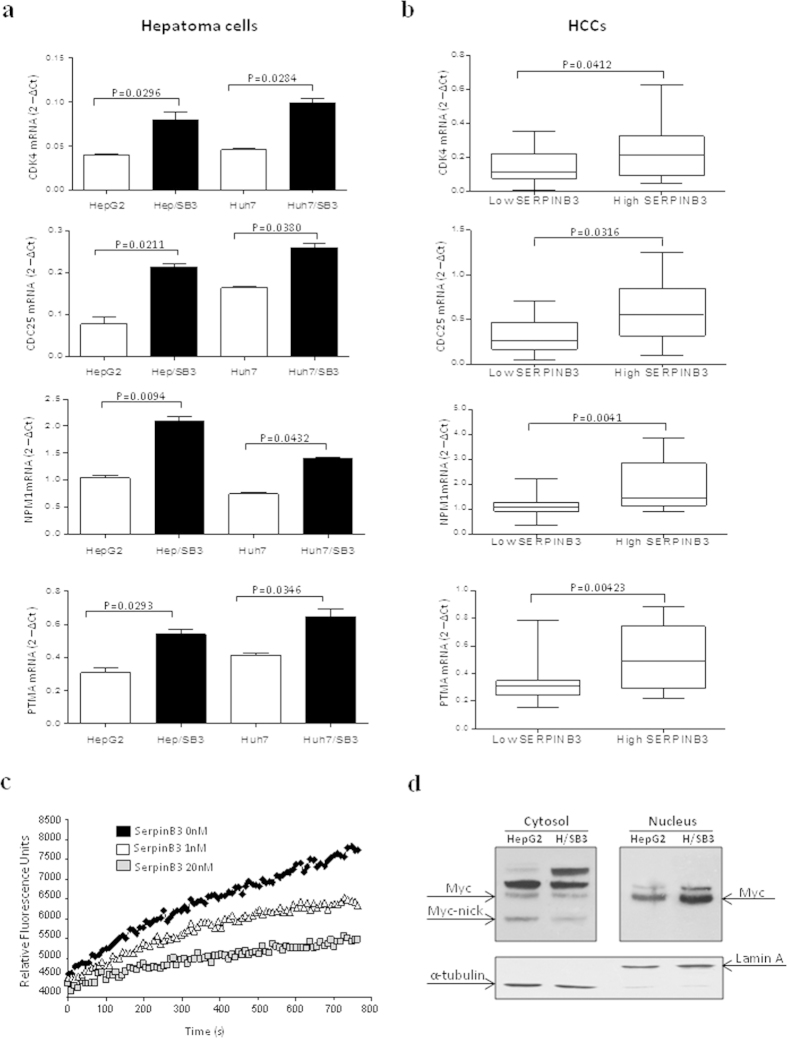
SerpinB3 modulatory effects on Myc target genes, calpain and cytoplasmic Myc-nick. (**a**) Quantitative mRNA analysis of the Myc target genes CDK4 and CDC25A in control HepG2 and Huh7 cells and in the corresponding cells transfected with SerpinB3 (Hep/SB3 and Huh7/SB3). Values represent normalized mean ± SEM of three independent experiments performed in triplicate. (**b**) mRNA expression of the same Myc target genes in human HCC specimens, in relation to low (<median value) SerpinB3 expression (N = 52) or high (>median value) SerpinB3 expression (N = 15). (**c**) The inhibitory effect of SerpinB3 on Calpain was examined using the commercially available Calpain activity assay Kit (Calbiochem, Merk Millipore, Darmstadt, Germany) that utilizes the Suc-LLVY-AMC as synthetic substrate. The release of the fluorescence product was monitored recording the fluorescence emission at 460 nm using an excitation wavelength of 360 nm with the Victor X3 multilabel plate reader (Perkin Elmer). The raw fluorescence data were expressed as relative fluorescence units (rfu). Inhibition experiments were performed using different amounts of SerpinB3 (range 0–20 nmol/L). (**d**) Modulatory effect of SerpinB3 on Myc-nick. Representative western blot protein analysis of Myc-nick on total extracts in control HepG2 and in HepG2 transfected with SerpinB3 (H/SB3) carried out with a specific antibody (anti-Myc N262) that recognizes the N-terminal portion of Myc.

In order to investigate whether the overexpression of Myc promoted by SerpinB3 was associated with an increased pro-oncogenic activity, we evaluated mRNA levels of four Myc target genes, including cyclin-dependent kinase 4 (CDK4), directly upregulated after Myc association with MAX, CDK activating phosphatase CDC25A^35^,^36^, Prothymosin-alpha (PTMA) and Nucleophosmin (NPM1)^37^. As expected, CDK4, CDC25A, PTMA and also NPM1 were up-regulated in SerpinB3 transfected cells (Fig. 5a). These features were confirmed in human HCCs, where a significant up-regulation of Myc target genes was detected in HCCs with high SerpinB3 levels, as compared to human specimens with low SerpinB3 levels (Fig. 5b).

### Modulatory effect of calpain

In order to investigate the possible mechanism responsible for nuclear translocation of Myc in cells expressing SerpinB3, we have explored whether this serpin may act through its interaction with Calpain. This hypothesis relies on the recent finding indicating that Calpain (i.e., a cysteine protease) can cleave Myc in the cytoplasm^14^ and might represent a putative target for the cysteine protease inhibitor SerpinB3. Accordingly, a dose dependent reduction of Calpain activity was determined by employing recombinant SerpinB3, with a marked inhibition obtained at 20 nmol/L (Fig. 5c).

### Modulatory effect of SerpinB3 on Myc-nick protein

In order to assess whether the amount of Myc-nick was modulated by SerpinB3, cytoplasmic Myc-nick was evaluated in HepG2 and HepG2/SB3 cells using a specific antibody that recognizes the N-terminal portion of Myc (anti-Myc N262)^14^. The positivity of Myc-nick was observed only in cytoplasmic fractions and was more evident in HepG2 cells than in SerpinB3 transfected cells, while it was undetectable in nuclear fractions (Fig. 5d). In addition, the obtained results confirmed the increase of nuclear Myc protein levels in SerpinB3 transfected cells versus HepG2 control cells.

Our findings suggest that calpain inhibition by SerpinB3 may modulate the balance of cytoplasmic/nuclear Myc by promoting its nuclear translocation as a result of an hampered production of Myc-nick.

### Modulatory effects of SerpinB3 on Yes-associated protein (Yap)

In order to better understand the mechanism underlying the transcriptional overexpression of Myc promoted by SerpinB3, we have hypothesized a possible involvement of the Yes-associated protein (Yap), a master oncogene commonly found up-regulated in the majority of HCCs.

**Figure 6:**
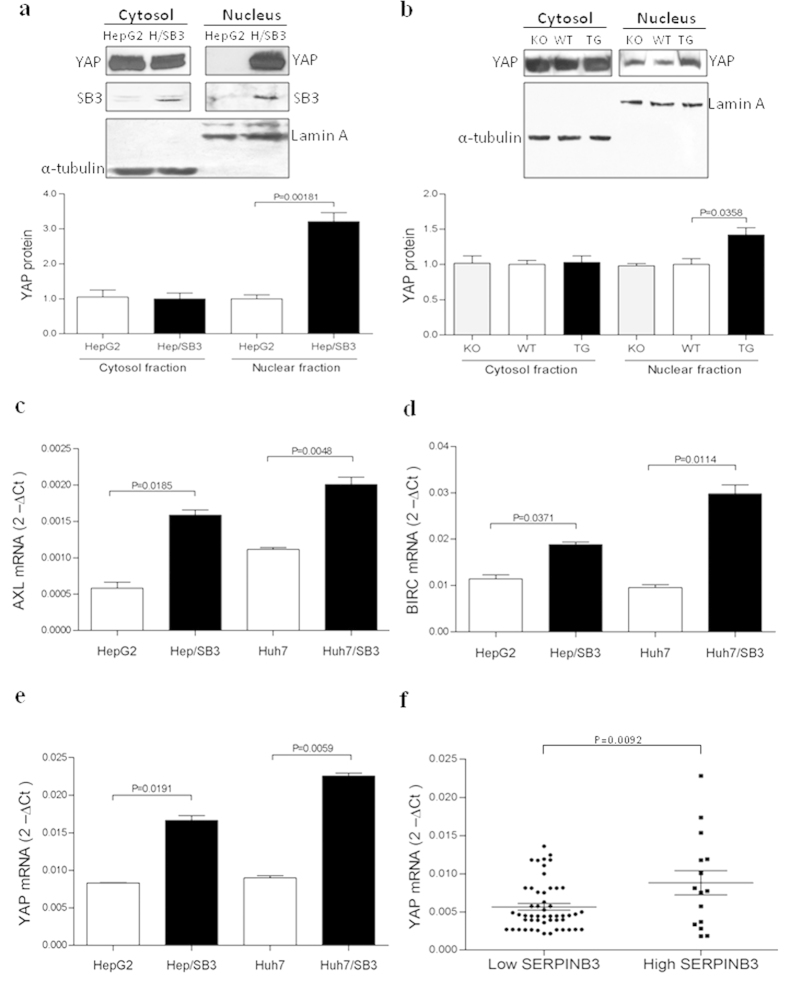
Modulatory effect of SerpinB3 on Yes-associated protein (Yap). (**a**) Example of Western blot analysis and corresponding densitometric analysis of Yap protein and its corresponding housekeeping bands performed on cytoplasmatic and nuclear extracts in control HepG2 and in HepG2 transfected with SerpinB3 (H/SB3). The corresponding densitometric analysis obtained in three distinct experiments of Myc subcellular localization. (**b**) Example of Western blot analysis and corresponding densitometric analysis performed in SerpinB3a(−/−)mice (KO); Wild type C57BL/6 mice (WT) and mice transgenic for human SerpinB3 (TG). The corresponding densitometric analysis was obtained in three distinct experiments and the cropped gels have been run under the same experimental conditions. (**c,d**) Quantitative mRNA analysis of Yap target genes AXL and BIRC5 in control HepG2/Huh7 cells and the corresponding SerpinB3 transfected cells (Hep/SB3 and Huh7/SB3). (**e**) mRNA expression of Yap in control HepG2/Huh7 cells and the corresponding SerpinB3 transfected cells (Hep/SB3 and Huh7/SB3). (**f**) mRNA expression of Yap in human HCC specimens in relation to low (<median value, N = 15) or high (>median value, N = 52) SerpinB3 expression. All values represent mean normalized expression ± SEM of three independent experiments performed in triplicate.

In order to evaluate Yap activity *in vitro* and *in vivo*, we performed Western Blot analysis in transfected HepG2 cells and in transgenic mouse livers, both overexpressing SerpinB3. Protein analysis revealed a remarkable nuclear translocation of Yap, especially in transfected HepG2 cells, as compared to controls (Fig. 6a,b). The Serpinb3a(−/−) mouse livers did not show any significant change of Yap protein levels versus controls, as shown in Fig. 6b.

In order to confirm the increased pro-oncogenic transcriptional activity of Yap, induced by SerpinB3, the mRNA expression of the Yap target genes Survivin (or Birc5) and Axl receptor tyrosine kinase (AXL) was analyzed. HepG2 and Huh7 cells stably transfected to overexpress SerpinB3 revealed a significant up-regulation of these two target genes, as compared to control cells (Fig. 6c,d). Of relevance, Yap mRNA levels were increased not only in transfected hepatoma cells (Fig. 6e), but also in human HCCs specimens with high SerpinB3 (Fig. 6f).

### Modulatory effect of Yap siRNA on Myc expression in hepatoma cells

**Figure 7:**
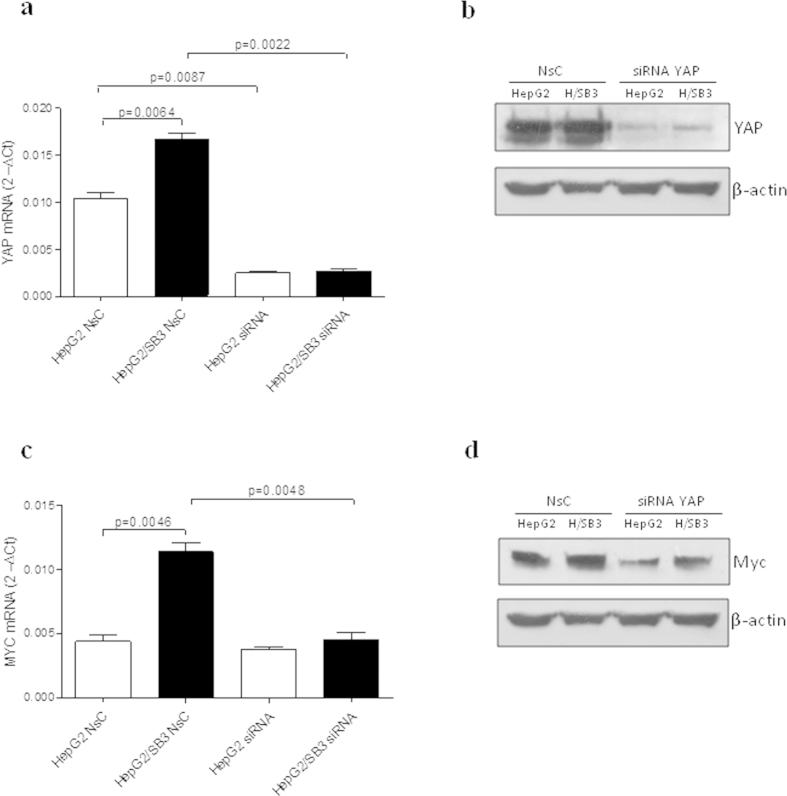
Effect of Yap siRNA on Myc expression in SerpinB3 cells. Representative quantitative mRNA analysis (**a**) and western blot protein analysis (**b**) of Yap on total extracts in control HepG2 and in HepG2 transfected with SerpinB3 (HepG2/SB3) silenced with siRNA Yap gene or transfected with non-silencing control (NsC). Representative quantitative mRNA analysis (**c**) and western blot protein analysis (**d**) of Myc on total extracts in control HepG2 and in HepG2 transfected with SerpinB3 (HepG2/SB3) silenced with siRNA Yap gene. Values represent mean gene expression  ± SEM.

To assess the functional impact of decreased Yap expression in the transcriptional modulation of Myc gene exerted by SerpinB3 in hepatoma cells, we knocked down Yap gene by lipofectamine transduction of siRNA. Real-time RT-PCR and immunoblot analysis confirmed down-modulation of Yap transcripts and protein at 72 h in both HepG2 and HepG2/SB3 cells (Fig. 7a,b).

Silencing of Yap determined also a remarkable reduction of Myc at mRNA and protein level (Fig. 7c,d) in HepG2/SB3 cells, suggesting that Yap may play an important role in promoting SerpinB3-induced transcription activity of Myc gene.

## Discussion

The precise modulation of Myc in the carcinogenesis process is still unclear. Among numerous potential oncogenic pathways, Myc has been observed to be a potent initiating oncogene for liver tumours and its inactivation is sufficient to induce sustained regression of Myc-initiated liver tumours in mice^38^. A frequent genetic abnormality seen in primary liver cancer is the overexpression of Myc. This oncogene assumes a pivotal role to determine HCC biology profile and a clinical value as predictor of poor outcome in clinical setting. Myc was identified as one of four genes, including Sox2, Octamer-4 and KLF4, that could reprogram cells to a pluripotent stem cell state^39^. Along these lines, we have recently reported that SerpinB3 is highly expressed in hepatic stem/progenitor cell compartment of both foetal and adult livers^40^. In addition, this serpin was found highly expressed in hepatoblastomas, a tumor originating from liver embryonic cells and once again SerpinB3 levels were positively correlated with Myc expression^33^.

We recently reported that SerpinB3 expression was up-regulated by hypoxia through HIF-2α-dependent mechanisms in human liver cancer cells^41^. HIF-2α has a great oncogenic capacity and its overexpression correlates with poor patient outcome in colorectal carcinoma, melanoma, ovarian cancer and hepatocellular carcinoma^42^,^43^,^44^,^45^, possibly also by promoting Myc activity through its binding to and stabilizing the Myc–Max heterodimer.

At variance with the Myc transgenic mouse model that is sufficient to induce liver cancer^46^, however, SerpinB3 transgenic mice do not develop any displasia or malignancies in the liver^47^. This discrepancy could be due to the fact that SerpinB3-overexpressing mice modulate only indirectly Myc gene, while the existence of other not yet discovered biological mechanisms determined by this serpin, that counteract Myc activity, at least from a phenotypic point of view, cannot be excluded.

The present study has been designed to investigate and characterize the interplay and functional modulation of Myc by SerpinB3 in human liver cancer. Here we provide for the first time evidence indicating that Myc expression is significantly and mechanistically up-regulated by SerpinB3 through calpain and Hippo-dependent molecular mechanisms in human liver cancer cells and in a transgenic mice model. The finding of significantly lower levels of Myc in the liver of SerpinB3a(−/−) mice further support the involvement of this serpin in the control of Myc expression. These findings were also confirmed in human samples of hepatocellular carcinoma, where the presence of high levels of SerpinB3 were correlated with higher expression of Myc and of Yap pathway activation.

From a biological point of view, Myc overexpression by itself is not sufficient to be considered as an equivalent of its pro-oncogenic activity. Several inhibitors, such as Calpain or let-7 family, could in fact interfere at several levels with Myc, counteracting its onco-properties in the cell cytoplasm. Nuclear localization of Myc is required to promote target oncogene expression. In the present study we have demonstrated that the nuclear localization of Myc was strongly associated with the presence of SerpinB3, both in hepatocellular carcinoma and in different experimental models. Furthermore, the recombinant SerpinB3 protein was capable to inhibit the activity of Calpain *in vitro*, likely reducing its ability to cleave Myc in its cytoplasmic form.

**Figure 8:**
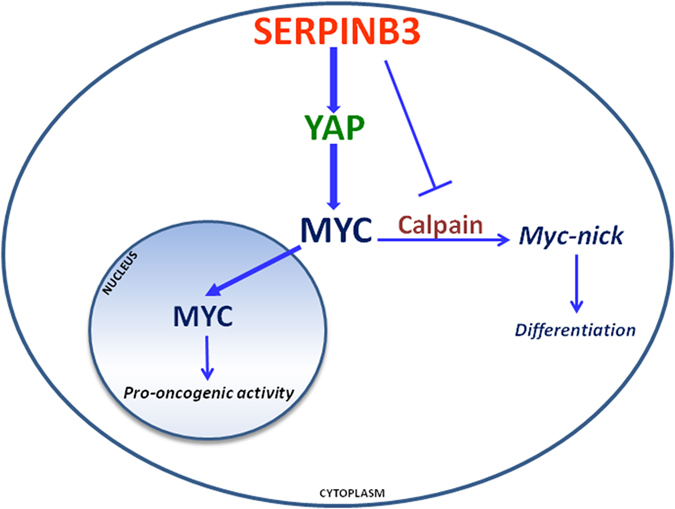
Hypothetical model of Myc modulation by SerpinB3. Proposed model depicting two strategies adopted by SerpinB3 to modulate Myc expression. 1. SerpinB3 determines an increase of Yap and its nuclear translocation, which can indirectly promote the increase of Myc transcription. 2. SerpinB3 blocks the Calpain-induced cleavage of Myc that determines its cytoplasmic retention, allowing the nuclear translocation of uncut Myc forms.

The novelty of our findings is that SerpinB3 can improve the activity of Myc adopting different strategies (Fig. 8). The first one is that intracellular SerpinB3 prevents Myc cytoplasmic retention, allowing its nuclear translocation, possibly blocking the cleavage exerted by Calpain that generates the non-oncogenic cytoplasmic Myc-nick form. Next, SerpinB3 is able to indirectly increase the transcription of Myc gene, probably through the induction of Yap pathway. Other mechanisms, however, cannot be excluded (e.g. the modulation of miRNA system) and deserve further studies. According to a recent report^48^, the action exerted by Yap on the miRNA-processing machinery is consistent with the miRNA profile induced by SerpinB3 in hepatoma cells^49^. The precise signal cascade leading to Myc overexpression still remains elusive and the biological influence of SerpinB3 on Yap and Hippo pathway should be further explored in detail by future studies.

In conclusion, the presented data provide further insights to our actual knowledge on the fine control of Myc modulation, possibly allowing the development of specifically designed and targeted drugs that may be able to more efficiently affect or prevent liver carcinogenesis.

## Materials and Methods

### Cell lines and culture conditions

HepG2 and Huh7 cells, derived from human hepatoma cells (LGC Standards S.r.l., Sesto San Giovanni, MI, Italy), were used. These cells were stably transfected with a plasmid vector carrying the wild-type SerpinB3 human gene (/SB3) or with the plasmid vector alone (pcDNA3.1D/V5-His-TOPO, Invitrogen Life Technologies, NY, USA), as previously reported^28^.

### Mouse models

The study has been carried out on (12–14 weeks old) C57BL/6 mice transgenic for human SerpinB3 (N = 4), whose initial colony was kindly provided by Prof. Cassani (Tecnogen, Caserta, Italy)^50^. Wild type C57BL/6 mice (N = 6) of similar age were used as controls. Serpinb3a knockout mice (N = 3) (kindly provided by Dr. Gary Silverman and Dr. Cliff J. Luke, University of Pittsburg, Children’s Hospital, Pittsburg, PA) have been used as additional negative controls. All animals were kept under specific pathogen-free conditions and maintained with free access to pellet food and water at the Animal Care Facility of the Experimental Surgery Division of the University of Padua.

Animals experiments, approved by the local Ethical committee and by the Italian Ministry of Health, were conducted in accordance with the Principles of laboratory animal care (NIH publication no. 85–23, revised 1985; http://grants1.nih.gov/grants/olaw/references/phspol.htm).

### Patients and samples

In the present study we employed HCC specimens, obtained under written informed consent at the time of surgery in 67 patients with underlying cirrhosis of different etiology.

Clinical-pathological and molecular characteristics as well as follow-up procedures applied to these patients were described in a previous study^32^. All the patients underwent surgical resection as first-line therapy without pre-operative anticancer treatment and distant metastases. Tumour tissue samples were collected and part was formalin fixed and paraffin embedded, whereas the remaining part was immediately frozen at −80 °C for transcript analysis. The use of human material conforms to the ethical guidelines of the 1975 Declaration of Helsinki and all experimental protocols were approved by the Ethical Committee of our Institution.

### Immunohistochemistry

Immunohistochemistry (IHC) was performed on formalin-fixed, paraffin-embedded specimens obtained from tumor liver samples as well as, from the liver of SerpinB3 transgenic mice and related wild type mice. Briefly, paraffin sections (2μm thick), mounted on poly-L-lysine coated slides, were incubated with primary antibody raised against SerpinB3 (1:1000, ThermoFisher Scientific, IL, USA on mouse liver tissue and 1:50, sc-21767 Santa Cruz Biotechnology, CA, USA on human liver tissue) and c-Myc (1:150, sc-788 Santa Cruz Biotechnology, CA, USA). After blocking endogenous peroxidase activity with 3% hydrogen peroxide and performing microwave antigen retrieval, primary antibodies were labeled by using EnVision, HRP-labeled System (DAKO) and visualized by 3′-diaminobenzidine substrate. For negative controls the primary antibodies were replaced by isotype- and concentrations-matched irrelevant antibody. Images of representative fields were captured by Leica Qwin Plus v3 software, under a CCD camera connected to a Leica microscope (Microsystem Imaging Solutions Ltd.).

### Western Blot analysis

Total cell lysates or nuclear vs cytosolic extracts were subjected to sodium dodecyl sulfate-polyacrylamide gel-electrophoresis on acrylamide gels, incubated with desired primary antibodies, then with peroxidase-conjugated anti-mouse or anti-rabbit immunoglobulins in Tris-buffered saline-Tween containing 2% (w/v) non-fat dry milk and finally developed with the ECL reagents, according to manufacturer’s instructions. Western Blot analysis was carried out in hepatoma cells transfected with SerpinB3 and in transgenic/knockout mice. The following specific antibodies were used: anti-SerpinB3 polyclonal antibody (1:500 Xeptagen SpA, Marghera, VE, Italy); anti-Myc monoclonal antibodies (1:1000, MYC clone 9E10, Santa Cruz Biotechnology, CA; anti-Myc polyclonal antibody (1:1000, clone N-262, SC-764, Santa Cruz Biotechnology, CA), anti-Myc polyclonal antibody (clone C-19, SC-788, Santa Cruz Biotechnology, CA), anti-YAP polyclonal antibody (1:200, YAP clone H-125, Santa Cruz Biotechnology, CA). Sample loading was evaluated by reblotting the same membrane with β-actin (1:1000, Sigma Aldrich) for total extract and α-tubulin (1:1000, Sigma Aldrich) or anti Lamin A (1:1000, SC-20680 Santa Cruz Biotechnology) for cytosolic or nuclear fraction respectively.

### Immunofluorescence

Indirect immunofluorescence was performed on HepG2 and HepG2/SB3 cells seeded on 6-well culture plates as described. Briefly, cells were fixed with methanol:acetone 1:1 (v/v) for 20 min at −20 °C, permeabilized with PBS containing 0.5% Triton X-100 and 0.05% NaN_3_ for 10 min (room temperature) and incubated with primary antibodies SerpinB3 (1:100, ThermoFisher Scientific) and c-Myc (1:100, sc-788 Santa Cruz Biotechnology, CA) overnight at 4 °C. Immunopositivity was revealed by means of appropriate Cy3-conjugated antibodies (1:1000 dilution). Nuclei (blue fluorescence) were stained by treating cells with 4,6-diamidino-2-phenylindole (DAPI, 1 mg/ml in methanol) for 30 min at room temperature. Slides were then mounted with ELVANOL (Sigma-Aldrich, St. Louis, MO) and observed under fluorescence microscope (Leica).

### mRNA quantification

Total RNA was extracted using RNasy Trizol (Invitrogen, Carlsbad, CA) according to the manufacturer’s instructions. After determination of the purity and the integrity total RNA, complementary DNA synthesis, quantitative real-time PCR reactions (RT-PCR) were carried out as previously described^32^ using the CFX96 Real-Time instrument (Bio-Rad Laboratories Inc, Hercules, CA, USA).

In hepatoma cells and HCC samples the relative expression were generated for each sample by calculating 2-ΔCt^51^.

The following sets of primers were used: MYC-F: 5′-aagacagcggcagcccgaac-3′; MYC-R: 5′-tgggcgagctgctgtcgttg-3′; CDK4-F: 5′-cagtgtacaaggcccgtgat-3′; CDK4-R: 5′-cagtcgcctcagtaaagcca-3′; CDC25A-F: 5-aggagtctccacctggaagac-3′; CDC25A R: 5′-ccattcaaaacagagccataa-3′; AXL-F:5-gcaggctgaagaaagtccct-3′; AXL-R: 5′-ctgtccatcccgaagccaat-3′; BIRC-F: 5′-aggaccaccgcatctctacat-3′; BIRC-R: 5′-aagtctggctcgttctcagtg-3′; YAP-F: 5′-tcagacaacaacatggcagga-3′; YAP-R: 5′-ttcatggctgaagccgagtt-3′; SerpinB3-F: 5′-aactcctgggtggaaagtcaa-3′; SerpinB3-R: 5′-accaatgtggtattgctgccaa-3′; NPM1-F: 5′-ggttgtgaactaaaggccga-3′; NPM1-R: 5′-cctttgcaccagcccctaaa-3′; PTMA-F: 5′-aaggagaagaaggaagttgtgga-3′; PTMA-R: 5′-ctacctcattgtcagcctcctg-3′.

### Calpain inhibition assay

The inhibitory effect of SerpinB3 on calpain was examined using the commercially available Calpain activity assay Kit (Calbiochem, Merk Millipore, Darmstadt, Germany) that utilizes the Suc-LLVY-AMC as synthetic substrate. Inhibition experiments were performed using different amounts of human recombinant SerpinB3 protein^52^ (range 0–20 nmol/L). 60μl of human calpain 1 (100 nmol/L) was mixed with increasing amounts of inhibitor (SerpinB3) in presence of 60μl of substrate at 1:100 dilution. Calpain activation buffer containing Ca^2+^ and the reducing agent TCEP (Tris 2-carboxyethyl phosphine hydrochloride) were added to the reaction mixture, to obtain a final volume of 230μl. The reaction was started by rapidly mixing of calpain in the reaction mixture and the formation of the fluorescent product was monitored over time. The release of the fluorescence product (AMC) was monitored recording the fluorescence emission at 460 nm using the Victor X3 multilabel plate reader (Perkin Elmer) at the excitation wavelength of 360 nm. The raw fluorescence data were expressed as Relative Fluorescence Units (RFU).

### YAP silencing by small RNA interference

RNA interference experiments to knockdown Yap expression in HepG2 cells were performed using siRNA duplex (Qiagen Italia, Milano, Italy) as previously described^53^. The following target sequences were used: YAP F: 5′-GGUGAUACUAUCAACCAAATT-3;

YAP R: 5′-UUUGGUUGAUAGUAUCACCTG-3′. The siRNAs and related non-silencing controls were transfected in HepG2 cells and in HepG2/SerpinB3 with Lipofectamine 2000 transfection reagent (Life Technologies Italia, Monza, Italy) according to manufacturer’s instructions up to 72 hrs.

### Statistical analysis

Data in bar graphs represent means ± SEM and were obtained from average data of at least three independent experiments performed in triplicate. Luminograms and morphological images are representative of at least three experiments with similar results. Statistical analysis for these experiments was performed by Student’s t-test (and Nonparametric test) or ANOVA for analysis of variance when appropriate. All tests were two-sided. Clinical significance of Myc in tumours samples was assessed by analyzing its expression in relation to time to recurrence, where early recurrence was defined as HCC reappearance within 12 months from radical resection. Kaplan–Meier curves were performed according to Myc expression in patients with high (>median value) expression of Myc compared with patients with low levels of Myc and statistical analysis was performed using log-rank (Mantel–Cox) test. Univariate and multivariate logistic regression analysis were carried out to identify clinical and/or molecular predictors of early tumor recurrence. For multivariate analysis only variables achieving a significant result in univariate analysis were included. For molecular variables a cut-off value > median value was defined as “high-mRNA expression” to be considered as a dichotomous categorical variable, compared to the remaining cases, defined as “low- mRNA expression” as previously described^32^. The calculations were carried out with Graph Pad InStat Software (San Diego,CA) and SPSS 21.0 software (SPSS Inc, Chicago,IL). The null hypothesis was rejected at p < 0.05.

## Additional Information

**How to cite this article**: Turato, C. *et al.* SerpinB3 and Yap Interplay Increases Myc Oncogenic Activity. *Sci. Rep.* **5**, 17701; doi: 10.1038/srep17701 (2015).

## Supplementary Material

Supplementary Information
