# Supplementary material for: SerpinB3 and Yap Interplay Increases Myc Oncogenic Activity
Source: Sci Rep. 2015 Dec 4;5:17701. doi: 10.1038/srep17701 (PMC4669520; doi:10.1038/srep17701)
Supplement: Supplementary Information [file srep17701-s1.pdf]

Supplementary information

SERPINB3 AND YAP INTERPLAY INCREASES MYC ONCOGENIC ACTIVITY

[1]Cristian Turato, [2]Stefania Cannito, [1]Davide Simonato, [1]Gianmarco Villano, [2]Elisabetta Morello [1]Liliana Terrin, [1]Santina Quarta, [1]Alessandra Biasiolo, [1]Mariagrazia Ruvoletto, [1]Andrea Martini, [1]Silvano Fasolato, [3]Giacomo Zanus, [3]Umberto Cillo, [1]Angelo Gatta, [2]Maurizio Parola, [1]Patrizia Pontisso.

[1]Dept. of Medicine, University of Padova , [2]Dept. of Clinical and Biological Sciences, Unit of Experimental Medicine and Interuniversity Center for Liver Pathophysiology, University of Torino, [3]Unit of Hepatobiliary Surgery and Liver Transplantation, University of Padova, Italy

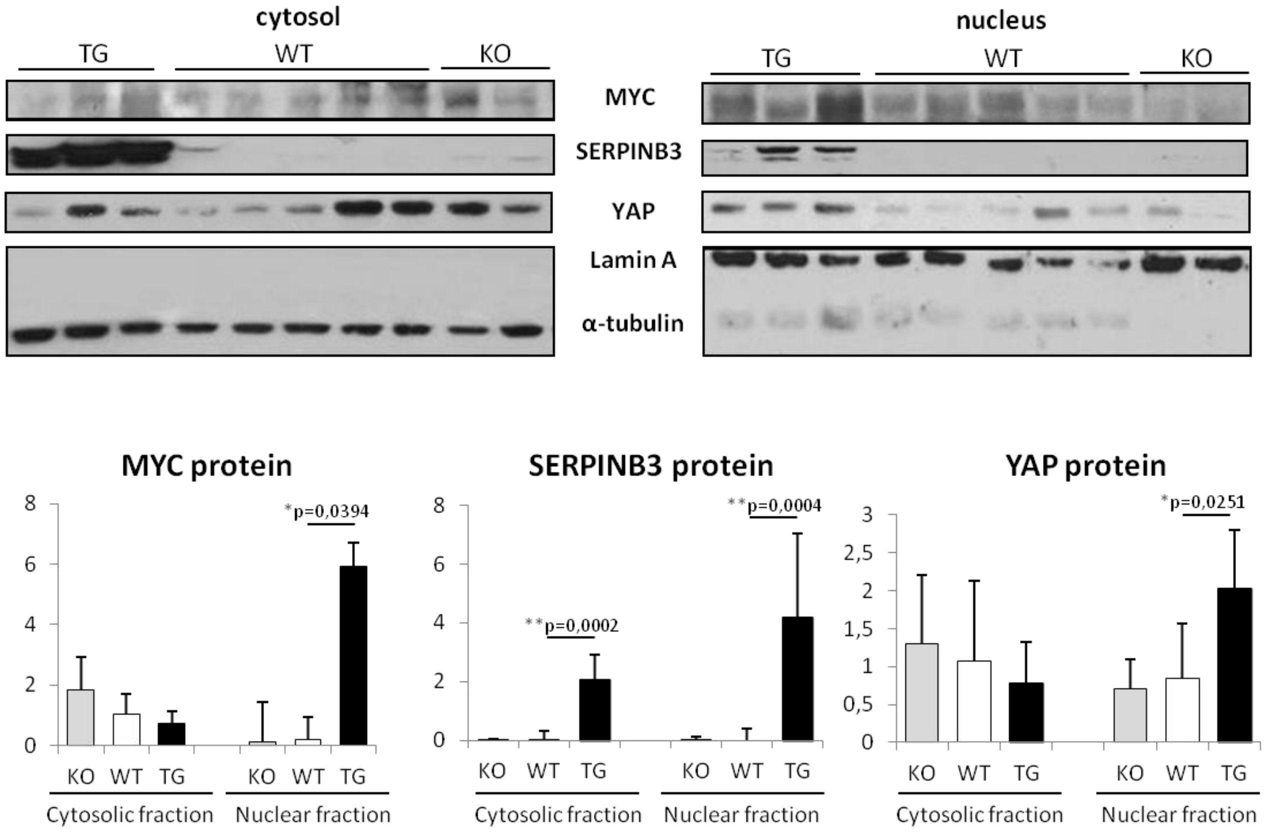

Supplemental Figure 1

Western blot analysis and corresponding densitometric analysis performed in SerpinB3a(-/-)mice (KO); Wild type C57BL/6 mice (WT) and mice transgenic for human SerpinB3 (TG). The cropped gels have been run under the same experimental conditions. Values represent mean protein expression ± SD. *Unpaired t test analysis.

VARIABLE	Univariate Analysis		Multivariate Analysis	
	p*	OR (CI 95%)	p**	OR (CI 95%)
High SERPINB3	0.025	3.91 (1.19-12.87)	0.024	4.09 (1.20-13.99)
High MYC	1.000	1.08 (0.41-2.84)		
CHILD-PUGH Class (B-C)	0.04	3.81 (1.05-13.75)	0.041	4.01 (1.06-15.20)

Supplemental Table 1

Univariate and multivariate analysis of biological and clinical variables in relation to 12 months recurrence in patients with hepatocellular carcinoma.

OR, odds ratio; 95% CI, 95% confidence interval; High SerpinB3 (> median value); High Myc (>median value). *Fisher's exact tests; **Logistic regression analysis.
